# The Study of Homœopathic Philosophy*Bureau of Homœopathics, Kansas State Homœopathic Society, 1896.

**Published:** 1896-04

**Authors:** W. A. Yingling

**Affiliations:** Emporia, Kansas


### THE STUDY OF HOMŒOPATHIC PHILOSOPHY.*

W. A. YINGLING, M. D., EMPORIA, KANSAS.

Study itself is an art. It is more than simple reading or desultory scanning. The novice scans, peruses, and may have the

* Bureau of Homœopathics, Kansas State Homœopathic Society, 1896.

memory to become verbose in parrot-like repetition or in bombastic opposition. The student masters the thought, masticates the subject-matter, digests the ideas, and assimilates the facts. Simple reading is not study. Any one can read; only the student studies. Study implies careful and sincere investigation; deep and broad research; profound and scientific consideration.

It is not presumed that the student has a previous comprehension of the subject he is studying. Light and comprehension come with knowledge. He is a seeker after truth, after light; his aim in study is to find out the right and to keep searching and cogitating until he has the comprehension of the matter under consideration. The first advance in any line of study may be slow and tedious. It usually is. The weak mind denies because it cannot understand. The strong mind holds in reserve its opinion till comprehension comes through careful thought. The man below mediocrity permits his prejudice to form an opinion, and his research is merely to bolster up and to seemingly verify the hasty decision. The true student is not hasty in his conclusions, and never permits his own ignorance to darken his mind or bias his research. The weak mind refuses light because it is not in accordance with preconceived ideas or past experience; because it is beyond easy comprehension. The strong mind is open to conviction and is willing to accept whatever is proven. The one refuses to sincerely test the matter proposed or to permit others to demonstrate the truth by experimentation; the other is eager for an honest and thorough investigation and demonstration. Practically, one says his opinions must be upheld; the other submits to the divine authority of right.

Reason is a creature of education; it is often led by prejudice. Some things are above reason; beyond its sphere till experience expands and broadens its power. Reason deals with that with which it is familiar. It cannot deal with that which is beyond its ken. The higher law being above the present faculty of comprehension, the reason is confused. Acquired knowledge by careful research is essential to *true* reason. The reason cannot grasp and hold before the judgment that which it does not comprehend. Cogitation and study and research bring light and comprehension, and then the reason can act upon the subject-matter. It is impossible for the judgment to be clear and accurate while the reason is walking in the dark, or hesitates because of the want of proper understanding. Reason always implies knowledge, and where there is no knowledge there can be no reason.

Understanding is not essential to belief. There are many things that we accept as truth, as facts, that we do not understand. Further research brings more knowledge and that enables us to comprehend. This proposition is exemplified in the life of every school boy. The knowledge which seems so simple to the advanced student is incomprehensible to the beginner. As the child stands at the threshold of his education the field before him seems dark and obscure. Weary months and years of close application are essential to the easy comprehension of the domain of philosophy and science and the more advanced branches of a collegiate course. At first it is plodding along, drudging, accepting by faith alone without comprehension, largely, until the faculty is expanded and the comprehension developed. In time the thought-glance accomplishes more, comprehends more, than weary weeks of close study did at first. Only the ignorant rejects because he does not understand.

All our faculties are creatures of development. The greatest scholar had, at one time to learn his A B C's. This is nature's plan. Development is the law. Babies have been born with full sets of teeth, with all colors of hair, but such a monstrosity as an educated baby has never gladdened the heart of even a homœopathic father—whether of the low or high potency pretension. Even the elements of any branch of knowledge are difficult of apprehension to the beginner. With due knowledge and mental training the most abstruse problems become easy of understanding. These faculties vary in different individuals, but the law of development or progression is applicable to every one.

The morally honest man does not *deny* without a reason and sufficient knowledge to judge intelligently. The infidel, to be an honest man, must be a sincere student of the Bible without bias or prejudice. He must not only read his Bible, but he must be capable of judging of its contents by a thorough knowledge of hermenuetics. He must read his Bible in the original to know the exact contents. He must be versed in all the arguments pro and con. In a word, he must have a reason for his denial. The one who has never sincerely and honestly read his Bible, much less investigated the grounds of its authenticity and credibility, cannot be a man of integrity and honesty of character if he deny its genuineness. The first requisite of a philosopher is sincerity and integrity of purpose. Prejudice and bias and the effect of early influences must be held subservient to the truth; they must be put aside. He must view the subject calmly, definitively, fully, and decide upon its own merits. That a claim is contrary to past supposition is a reason to question, but not to deny. The past supposition may be contrary to the facts, and based entirely upon the *want* of experience. When experience can be brought in to verify the reasonableness of a claim, that experience must be sincerely sought, for experience is demonstration when based upon true knowledge, and such demonstration is above reason.

The honest student will also receive and hold under judgment the teaching of any system he accepts until he proves it to be wrong. He will not refuse to believe merely because he cannot at first understand. His duty is to receive the postulates of the system, to candidly consider them, weigh all the evidence for and against, and come to a legitimate conclusion. If the present status of his mental condition will not enable him to comprehend the position taken by the teacher, his duty compels him to prosecute his investigation until he can grasp the idea and comprehend the teaching. The want of ability to comprehend is never a sufficient reason to reject. If it were, the mass of mankind would reject the bulk of all knowledge. What now seems impossible, will, after due consideration and investigation, seem simple enough and perfectly tenable. The fact that the majority of mankind holds a postulate untenable does not prove it so. The majority has been and will likely again be in the wrong, as in the case of Galileo. It is natural for the masses to reject that which is novel, or contrary to their common or vulgar experience. The true student, the honest investigator, holds his bias and prejudice in subjection and seeks the true light, even though it may be contrary to his past opinions and notions. Opinion is not argument; a preconceived notion is not evidence. Only the weakling permits doubt to darken his judgment.

I think all present will acknowledge this train of reasoning to be legitimate and correct. Now let each one apply it to the system of medicine as taught by Samuel Hahnemann, and which he, the author of the system, has called by the name of Homœopathy.

It will not be admissible for the sincere student to reject Homœopathy merely because it is contrary to his past notions or beyond the easy comprehension of his mind. Nor will he be sincere to reject it because it introduces new ideas and conceptions of medicine. We must remember that our past notions may all be erroneous, and that Hahnemann may be the medical Galileo combating the false opinion of the world. It is something new, contrary to past usage; hence we must approach its study with frankness and sincerity. We dare not reject anything simply because it is not in accordance with our preconceived ideas and notions. We are to approach its study like true philosophers. We are to prove all things and hold fast to that which is good, *and to do it in the manner directed by the teacher*. To apply his system in accordance with our prejudiced notions, or without due care, and then reject it, possibly denounce it, would be neither scientific nor honorable. While the true student is not to blindly receive all that is asserted, he is to be open to conviction.

The man or woman who accepts Homœopathy as a system of medicine, and professes to practice it as such, is compelled to receive the teachings of Hahnemann unless he prove them to be false. He must accept all the tenets unless proven false.

He must test the system as directed by the founder in all sincerity, and to do this he must first comprehend it. In consistency with his own honor, he cannot reject any part of the system unless he first proves it to be false, and he cannot prove it false unless he has so thoroughly investigated it as to apply it in strict accordance with Hahnemann's instructions. To administer remedies in alternation or in the crude form, or to prescribe on a pathological name, is not Hahnemannian, and those who thus test the tenets of Homœopathy, and necessarily fail because they have tested it by some other rule of practice than the law of homœopathic procedure, cannot consistently and fairly pronounce the teachings of Hahnemann to be at fault. The professed follower of Hahnemann to be consistent and fair in the renunciation of Homœopathy, in pronouncing Homœopathy a failure, must first fully comprehend its tenets and fairly apply them. Those who have best understood the teachings of Hahnemann, and fairly tested the doctrines of Homœopathy, realize more fully than any other class that every failure to cure a curable disease is owing more to the ignorance and inability of the prescriber than to the fault of the homœopathic law of cure.

*The Organon of the Healing Art* and *The Chronic Diseases* were written by Hahnemann for the purpose of teaching his system of medicine. They are the only original and sufficient source of information, and, being the basis of Homœopathy, it becomes absolutely necessary for every professed practitioner of the system to be a student of them. Yet how many there are who pronounce Homœopathy a failure in whole or in part, and declare Hahnemann to be visionary, who have never even read his basal works! How can any sincere man, any fair-minded man, who professes to practice Homœopathy, deny the teachings of Hahnemann till he has carefully studied *The Organon* and implicitly followed its teachings? Would it not be as consistent for the professed minister of the gospel to call Christ's religion a failure and His teachings erroneous when he did not even own a copy of the Bible, and had never read it, much less studied it? Or could such a minister pronounce the doctrine of Justification, or any other doctrine, wrong when he could have no proper conception of the doctrine? Such a minister would be considered a very poor representative of the religion of Christ.

The study of homœopathic philosophy is difficult in that it is contrary to common custom and practice. It makes a complete change from the crude material to the spirit-like power of drugs. It is also opposite in its application. The mind of men clings to the tangible and material, as well as to the familiar. It is not easy for him to make a change in opinion and practice, especially when the change implies so much. Yet there are Galileos and Hahnemanns who declare the new truth and prove by clear teaching and demonstration that the former opinions and practices have been erroneous. The world to-day acknowledges Galileo correct, and the time is hastening onward when Hahnemann will also be acknowledged the founder of the only true system of medicine and the homœopathic the only law of cure.

In conclusion, we would call attention to the fact that the greatest and most successful homœopathists were close students and followers of Hahnemann. Those who wrote the books that do us the most good, the living books, were constant readers of *The Organon.* The closer we follow the immortal Hahnemann the greater will be our success as physicians.

